# Local epigenomic state cannot discriminate interacting and non-interacting enhancer–promoter pairs with high accuracy

**DOI:** 10.1371/journal.pcbi.1006625

**Published:** 2018-12-18

**Authors:** Wang Xi, Michael A. Beer

**Affiliations:** 1 Department of Biomedical Engineering John Hopkins, Baltimore, MD, United States of America; 2 McKusick-Nathans Institute of Genetic Medicine, Johns Hopkins University, Baltimore, MD, United States of America; University of Washington, UNITED STATES

## Abstract

We report an experimental design issue in recent machine learning formulations of the enhancer-promoter interaction problem arising from the fact that many enhancer-promoter pairs share features. Cross-fold validation schemes which do not correctly separate these feature sharing enhancer-promoter pairs into one test set report high accuracy, which is actually arising from high training set accuracy and a failure to properly evaluate generalization performance. Cross-fold validation schemes which properly segregate pairs with shared features show markedly reduced ability to predict enhancer-promoter interactions from epigenomic state. Parameter scans with multiple models indicate that local epigenomic features of individual pairs of enhancers and promoters cannot distinguish those pairs that interact from those which do with high accuracy, suggesting that additional information is required to predict enhancer-promoter interactions.

## Introduction

While quantitative modeling of cell-specific enhancer activity and variant impact is progressing rapidly, predicting the promoter and gene targets of enhancers remains challenging. We therefore read with great interest the paper by Whalen, et al,[1] which reported a computational model (TargetFinder) using epigenomic features that could predict enhancer-promoter (EP) interactions with high accuracy. In this brief note we report that because many EP pairs share features, the random cross-fold validation scheme used in Ref. 1 fails to properly evaluate generalization error and produces inflated test set accuracy. Proper cross-fold validation schemes predict EP interactions with much lower accuracy, due to the fact that many of the models used exhibit significantly lower performance on a reserved test set than on the training set data. If a test set is not fully reserved, these classifiers will fail to generalize to data outside the training data.

## Results

**Fig 1:**
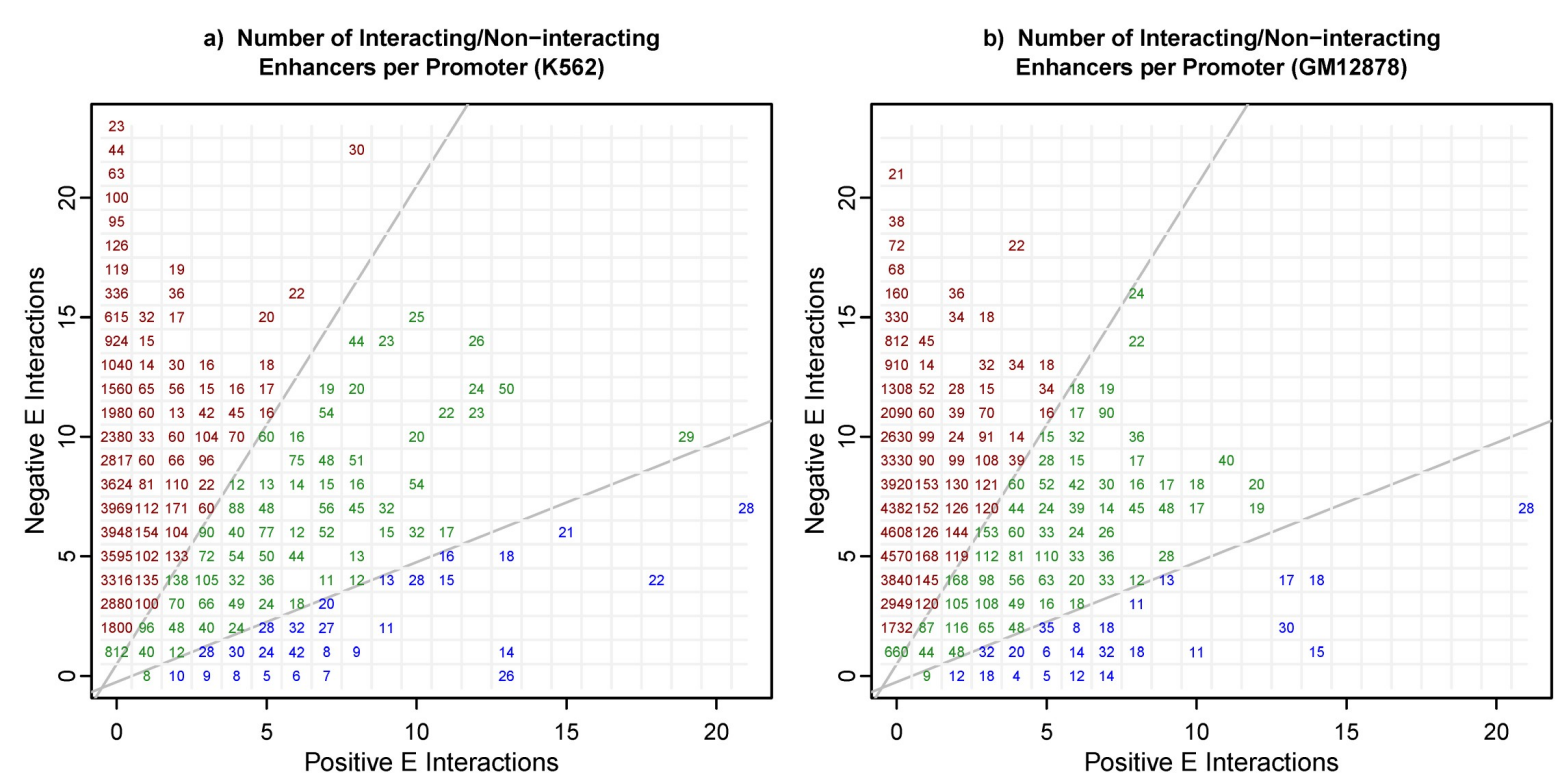
Most of the EP pairs used for training in Ref. 1 share promoters and promoter features with multiple EP pairs. **a)** For cell line K562, and **b)** for cell line GM12878, for each promoter we show the number of positive and negative EP pairs sharing a promoter. In **a)** for example, there are 27 interactions in position (7,2), which means three distinct promoters each interact positively with 7 enhancers and negatively with two enhancers in the training data. When the number of negative and positive EP pairs interacting with a promoter are imbalanced, they can be predicted correctly from training set promoter features. The blue interactions have a 2-fold or greater imbalance (pos>neg) and the red interactions have a 2-fold or greater imbalance (neg>pos), and both red and blue interactions (92% of the data) can lead to test set contamination by shared promoter features.

Whalen, et al,[1] used an F1-score performance measure, F1 = 2P∙R/(P+R), where P = precision and R = recall, and reported that their average F1 across 6 cell lines was 0.83. This typically implies P,R>0.8 and AUPRC,AUROC~0.9, and that most EP pairs are being correctly classified as interacting or not according the epigenomic state of the enhancer (E), promoter (P), and the intervening genomic interval, or window (W), between the enhancer and the promoter. Interestingly, they found that window features (W) were often selected as being most predictive in the model, and that lack of some epigenomic marks in the genomic interval were correlated with positive EP interactions, a compelling hypothesis. Curiously, this high performance was only achieved with gradient boosting using a very large number of trees, and not with a linear SVM, which achieved F1-score≈0.2. In the course of examining their training data to find direct evidence in support of this hypothesis, we noticed that partly due to the coarser resolution of the Hi-C interaction data, multiple contiguous enhancers often positively interact with the same promoter, but are all labelled independent positive EP interactions for training, even though they potentially share P and W features. If samples with identical features are not in the same cross-fold validation test set, these shared features could lead to incorrect evaluation of generalization performance. There are two distinct mechanisms by which features could be shared. The simplest is that many EP pairs share a promoter. In the K562 and GM12878 training data from Whalen, et al,[1] over 92% of EP pairs share a promoter with another EP pair and have a 2-fold or greater positive/negative class imbalance, and are thus subject to significant test set contamination through the shared P features. In Fig 1 we show how many EP pairs share a promoter with a given number of positive and negative enhancers (positive and negative EP pairs connected to a single promoter). For clarity, this plot is repeated in S1 Fig showing the number of promoters and shared enhancers, but since these models are trained on EP pairs, the EP pair counts in Fig 1 are relevant for understanding the degree of potential test set contamination. The greater the class imbalance, the more accurately this set of EP pairs can be predicted from training set promoter features alone. This is a serious problem for all pairs labelled red or blue in Fig 1. For example, at position (7,2) in Fig 1A, there are 27/(7+2) = 3 distinct promoters in the training data which each interact positively with 7 enhancers and negatively with two enhancers (two negative EP pairs) in the training data. When one of these positive pairs is in the training set, a specific but non-generalizable rule on that promoter’s features would predict the other pairs with accuracy of 78% when they are in the test-set. This potential problem becomes especially problematic for all EP pairs near the x and y axes in Fig 1, and most of the negative samples are particularly susceptible. The second mechanism by which EP pairs could share features is that the window (W) features are defined to be the epigenomic signals in the interval between the enhancer and promoter elements, so enhancers on the same side of the promoter can share window features, or more generally, any overlapping EP pair can share some common W signal features. We used cross-fold validation (CV) test sets sorted by chromosomal position so that no EP pairs with either P or W shared features could be in the same test set, and test set performance could be correctly evaluated. We also tested a cross-fold validation scheme where all EP pairs sharing a promoter were constrained to be in the same test set (promoter segregated). In this case, promoter features cannot lead to test set contamination, but window features can when using EPW features or W features to train the model.

**Fig 2:**
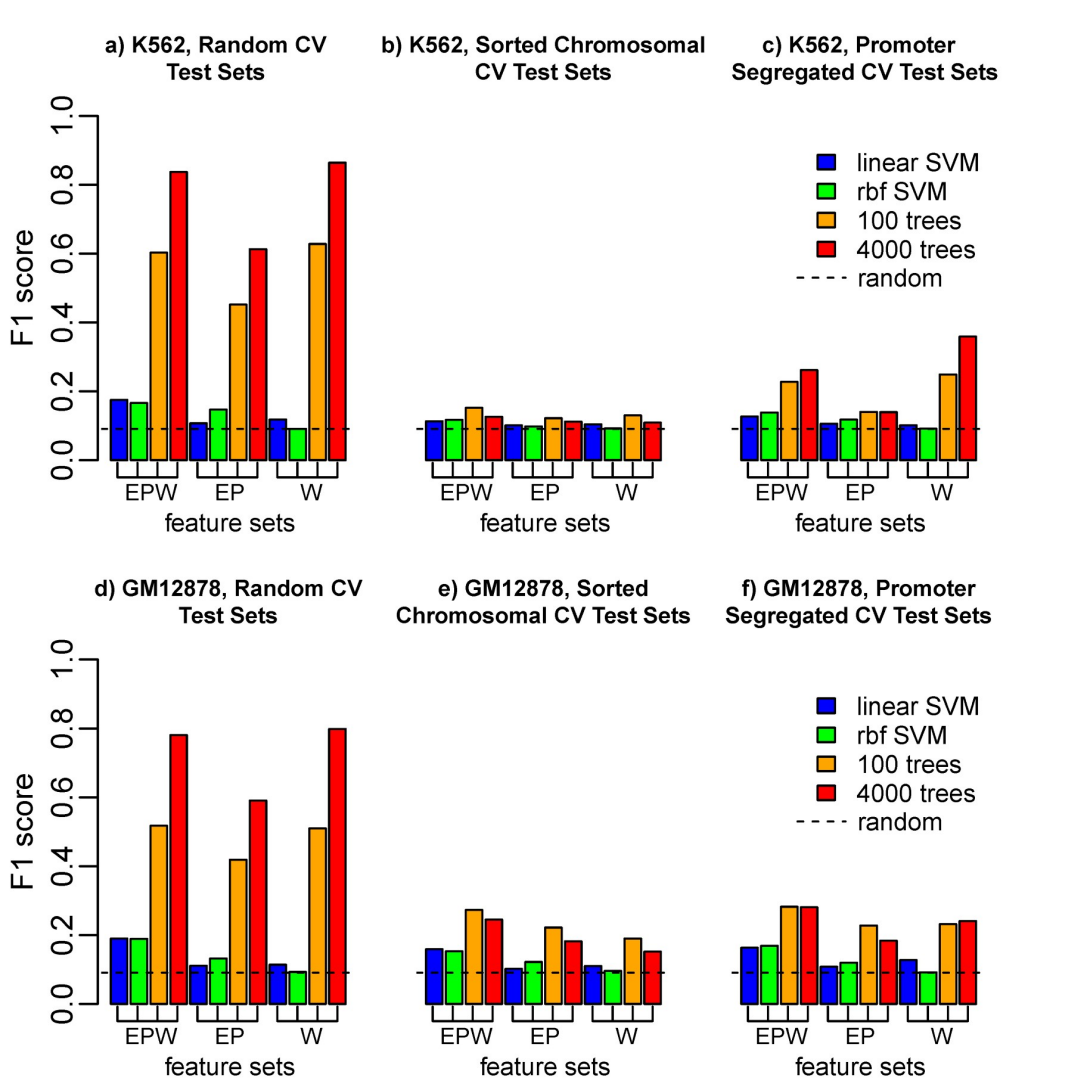
The apparently high predictive power of epigenetic state to identify enhancer-promoter (EP) interactions is largely due to incorrect evaluation of generalization performance. **a)** For cell line K562, using training data from Ref. 1, with random assignment of positive and negative EP pairs to cross-fold validation (CV) test sets, a gradient boosting classifier with a very large number of trees (4000) is able to achieve F1>0.8 with EPW or W features. **b)** If EP pairs with shared epigenomic features are properly forced to be in the same CV test set by sorting by chromosomal position, the predictive power is only slightly better than random, F1 = 1/11. **c)** If EP pairs sharing the same promoter are forced to be the same test sets, test set contamination through EP features is eliminated, but some can still occur through shared window features (W). **d,e,f)** A similar reduction in performance is observed when removing test set contamination in cell line GM12878 with sorted chromosomal CV test sets.

We reproduced the published results from TargetFinder using the training data for the K562 cell line as shown in Fig 2A), and when we further observed that a nonlinear SVM (rbf with default C and γ) could not achieve the high performance of gradient boosting, and that high test-set F1 is only achieved with 4000 trees, we suspected that test set contamination was important. When we instead used cross-fold validation (CV) test sets sorted by chromosomal position so that no EP pairs with shared features could be in the same test set, we found that test-set F1 dropped dramatically, as shown in Fig 2B. In fact, with sorted chromosomal CV test tests the predictive performance is only slightly better than random (F1 = 1/11 for this 1:20 ratio of positive to negative interactions). When using the promoter segregated CV test set scheme, P features cannot lead to test set contamination, but window features can, and do (EPW and W), when a gradient boosting classifier is used, Fig 2C. A similar but slightly less dramatic reduction in test-set predictive power is seen when removing or reducing the potential for test set contamination in the GM12878 cell line (Fig 2D, 2E and 2F).

**Fig 3:**
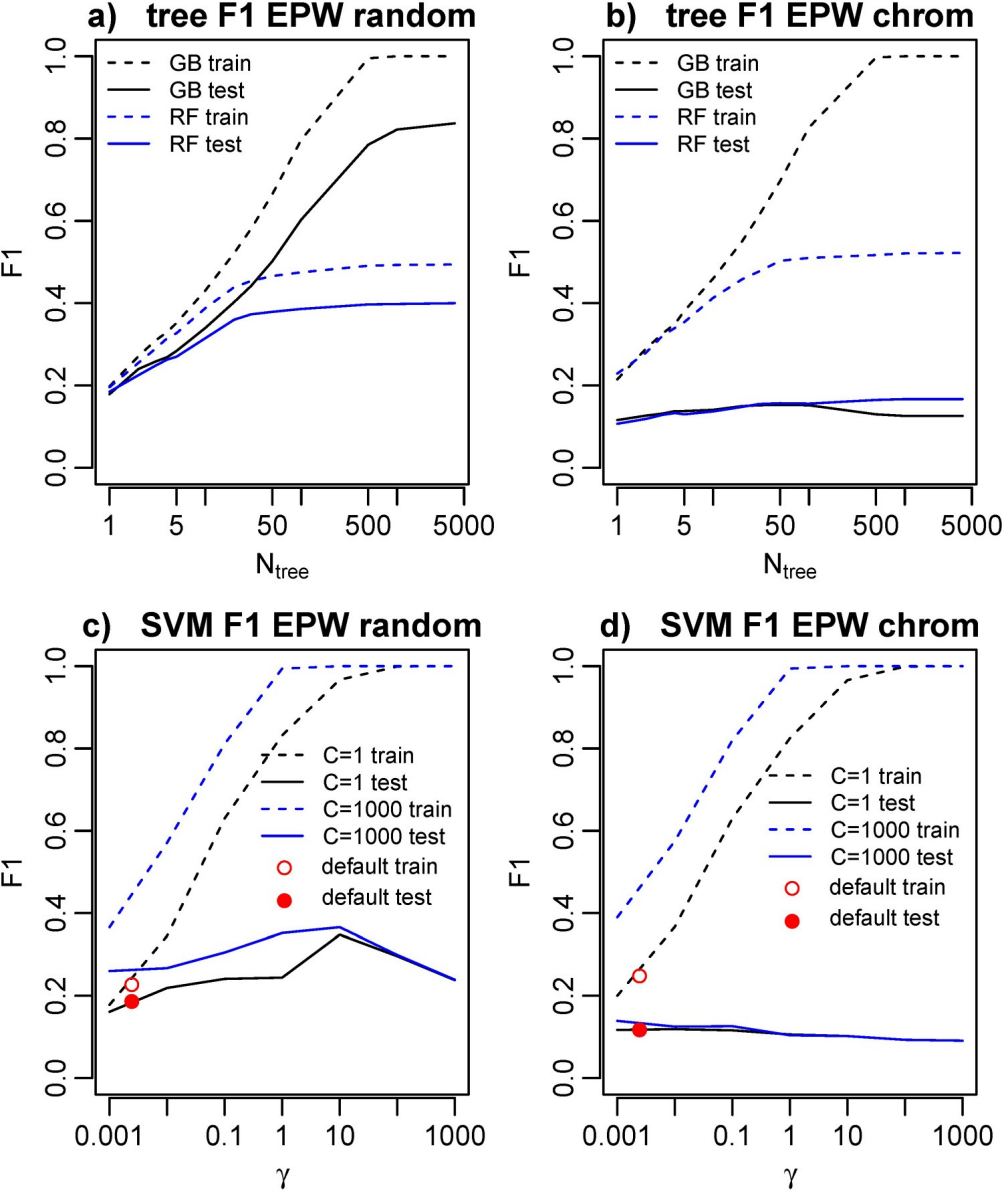
Parameter variation in gradient boosting (GB, black), random forest (RF, blue), and SVM models show significantly higher training set accuracy than test set accuracy for large number of trees or large C and γ. **a)** For cell line K562, with random CV test sets, test set performance appears high, but comparison with chromosomal CV test sets shows that this is due to test set contamination and failure to properly evaluate generalization performance. **b)** With chromosomal CV test sets, training set accuracy approaches 1 with large number of trees but now test set performance is correctly evaluated and test set F1 is very low. **c)** Nonlinear RBF SVM performance for incorrect random, and **d)** proper chromosomal CV test sets. For large γ, SVM training set accuracy approaches 1 and test set accuracy is low. For typical default RBF parameters C = 1 and γ = 1/n_features_ (red) test set performance is still not significantly better than random.

To examine generalization performance of these models detail, we directly compared training-set and test-set performance with multiple models, feature sets, and parameters, on both the random and chromosomally sorted CV test-set schemes. Test and training set F1 are shown in Fig 3A and 3B as the number of trees are varied for gradient boosting (used in Targetfinder) and random forest methods, using EPW features for K562. Training set F1 is much greater than test set F1 and increases with the number of trees. With random CV test sets this leads to an artificially inflated test set F1 = 0.84 for N_tree_ = 4000 (as reported in Ref. 1 and similar methods), while with chromosomal CV test sets the correct test set performance is F1 = 0.13. Full scans of training and test set AUROC, AUPRC, and F1 performance metrics for gradient boosting and random forests are shown in S2 Fig, showing high training set performance with large number of trees and low test set performance for all tree models with chromosomal CV test sets.

We next varied parameters for the nonlinear RBF SVM model. In the RBF SVM the key parameters are C, which controls the weighting for misclassification error, and γ, which describes the scale of the RBF kernel function and thus controls the smoothness of the decision boundary. For large C and γ the SVM is able to fit the training data with a convoluted decision boundary. The training and test set F1 of the RBF SVM are shown in Fig 3C and 3D for C = 1 and C = 1000 as γ is varied, and as expected, training set F1 approaches 1 for large γ. The default sklearn package defaults for the rbf SVM are C = 1 and γ=1/n_features_. For EPW, EP, and W feature sets n_features_ = (408, 272, and 136), so the default RBF SVM parameters are in the regime where there is little difference between training and test set accuracy (red points in Fig 3C and 3D). Full test set and training set performance for C = 1 and C = 1000 are shown in S3 Fig, and test set F1 for a complete C and γ grid scan is shown in S4 Fig for K562 and GM12878. SVM performance was slightly better for GM12878. Consistent with our results from the tree models, no choice of RBF SVM parameters or features yielded performance significantly better than random with proper chromosomal CV test sets.

## Discussion

We have shown that the high accuracy of TargetFinder[1] in predicting EP interactions is mostly due to improper evaluation of generalization performance. We consider it extremely likely that subsequent publications using similar or identical training data are also subject to this problem, and that reports of accurate predictive modelling of EP interactions from local epigenomic state should be re-evaluated.[2–4] On the bright side, there is now considerable room for improvement in models of EP interactions. Our results showing that no model performs much better than random guessing strongly suggests that local EP (and W) epigenomic state features alone are insufficient to distinguish interacting and non-interacting EP pairs. We suspect that EP interactions indeed may be specified by epigenomic state, but that additional features need to be considered, which are distinguishable from the feature set used in Ref 1 by their “non-local” nature. These additional features may include relative genomic position, the presence or absence of competition or interactions between E or P elements, conformational constraints, or subtle epigenomic state differences between enhancers competing for the same promoter or multiple promoters. Finally, we note that the inability to correctly evaluate test set performance is likely to be less of a problem in approaches which learn interactions between larger scale domains in non-overlapping chromosomal bins.[5]

## Supporting information

S1 Fig **a)** For cell line K562, and **b)** for cell line GM12878, we show the number of promoters with the specified number of positive and negative enhancers sharing a promoter. This is equivalent to Fig 1 but counts promoters instead of EP pairs.
(TIFF)

S2 Fig Training (dashed) and test set (solid) AUROC, AUPRC, and F1 performance for EPW, EP, and W feature sets and for random (top) and chromosomal (bottom) CV test sets using gradient boosting (GB, black) and random forest (RF, blue) methods, as number of trees is increased. Training set accuracy is significantly larger than test set accuracy for large numbers of trees, and test set performance is near random for all models with correctly segregated chromosomal CV test sets.
(TIFF)

S3 FigTraining (dashed) and test set (solid) AUROC, AUPRC, and F1 performance for EPW, EP, and W feature sets and for random (top) and chromosomal (bottom) CV test sets using a nonlinear RBF SVM model with C = 1 (black) and C = 100 (blue), as γ is varied.Training set accuracy is significantly greater than test set accuracy for large γ, and test set performance is near random for all feature sets with properly segregated chromosomal CV test sets.(TIFF)

S4 FigTest set F1 performance for nonlinear RBF SVM as C and γ are varied in complete grid parameter scan.For K562 performance is near random for chromosomal CV test sets for all parameter choices. For GM12878 F1 is only is slightly higher than random with chromosomal CV test sets.(TIFF)
