# Supplementary figures and images for: Local epigenomic state cannot discriminate interacting and non-interacting enhancer–promoter pairs with high accuracy
Source: PLoS Comput Biol. 2018 Dec 18;14(12):e1006625. doi: 10.1371/journal.pcbi.1006625 (PMC6298642; doi:10.1371/journal.pcbi.1006625)

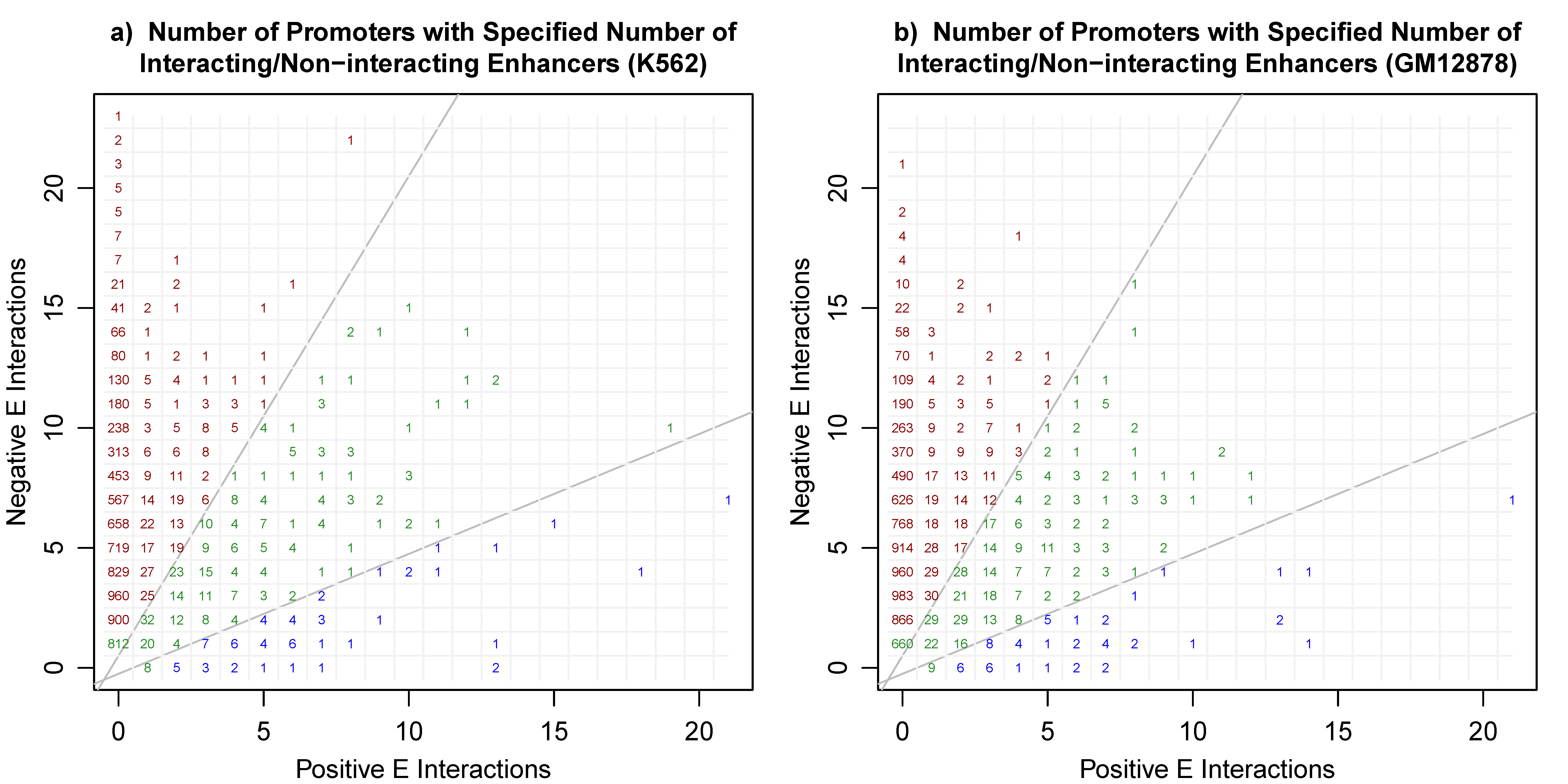

Supplement: S1 Fig — a) For cell line K562, and b) for cell line GM12878, we show the number of promoters with the specified number of positive and negative enhancers sharing a promoter. This is equivalent to Fig 1 but counts promoters instead of EP pairs. (TIFF) [file pcbi.1006625.s001.tiff]

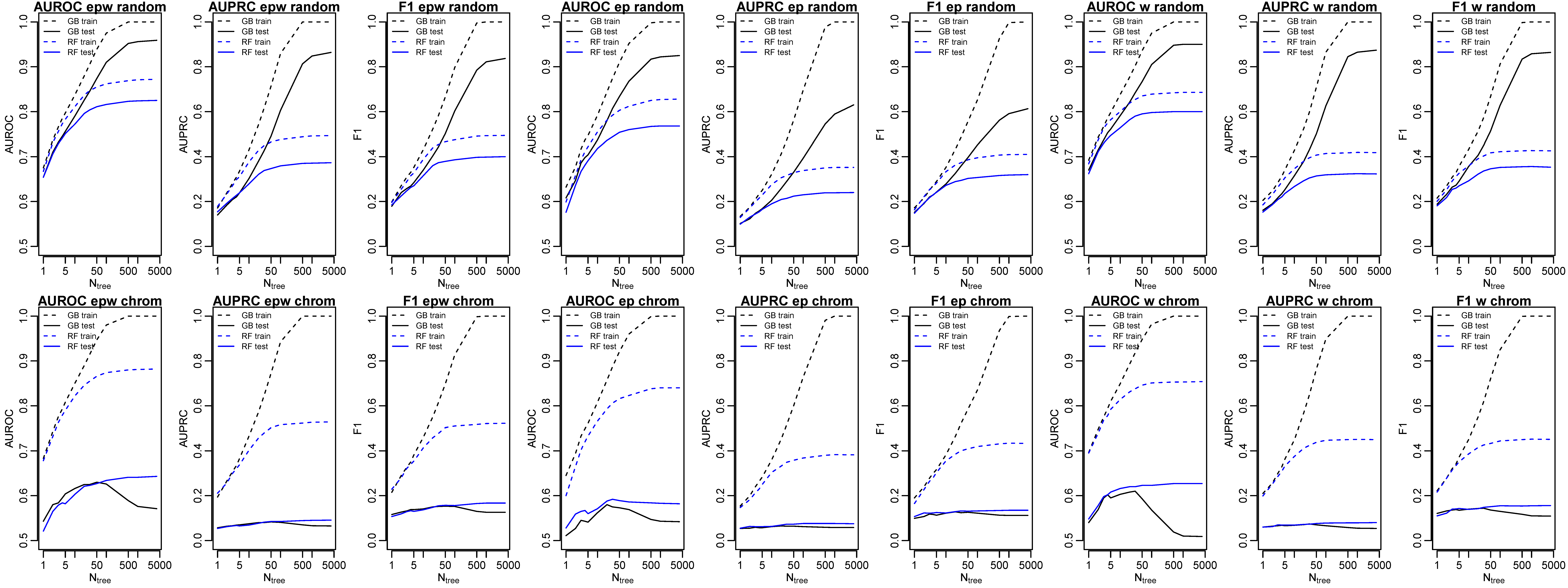

Supplement: S2 Fig — Training set accuracy is significantly larger than test set accuracy for large numbers of trees, and test set performance is near random for all models with correctly segregated chromosomal CV test sets. (TIFF) [file pcbi.1006625.s002.tiff]

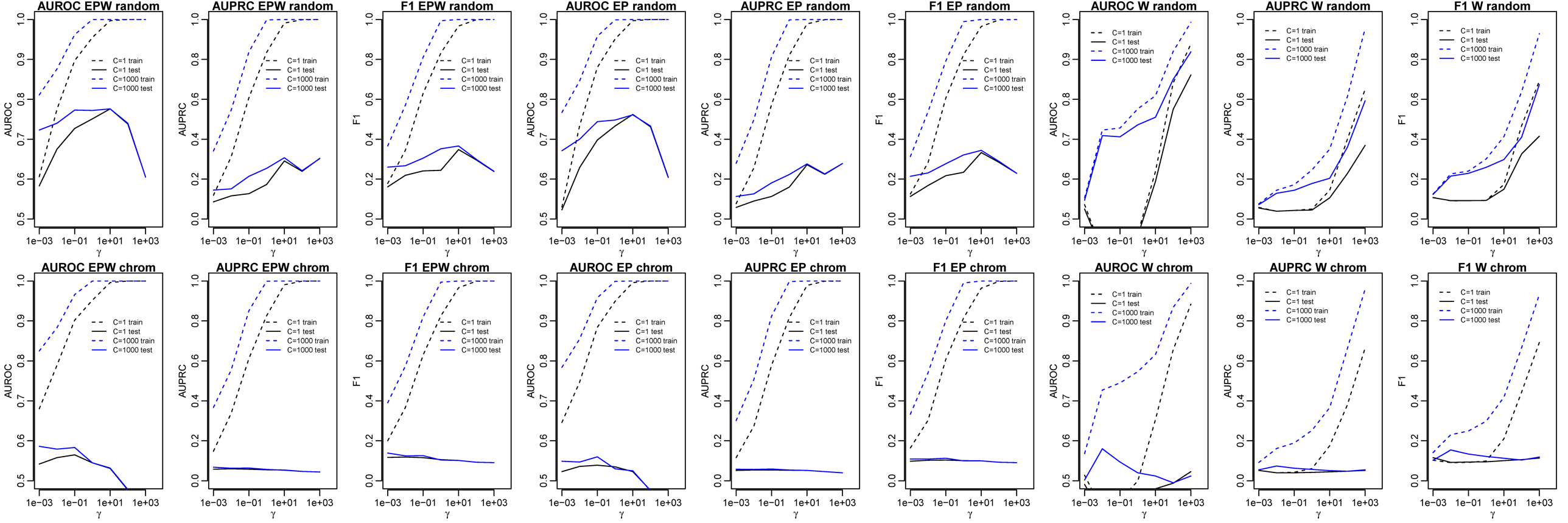

Supplement: S3 Fig — Training set accuracy is significantly greater than test set accuracy for large γ, and test set performance is near random for all feature sets with properly segregated chromosomal CV test sets. (TIFF) [file pcbi.1006625.s003.tiff]

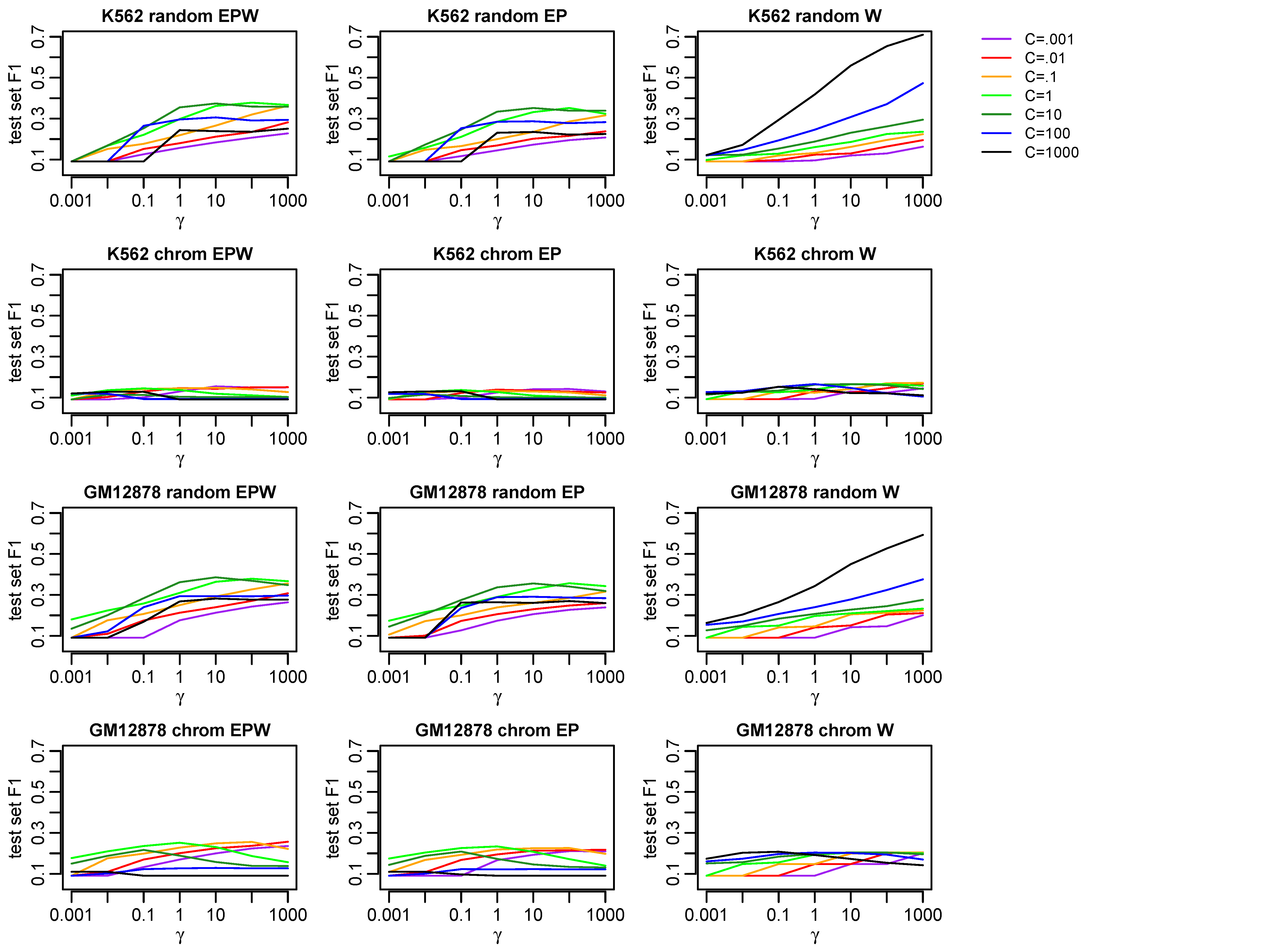

Supplement: S4 Fig — For K562 performance is near random for chromosomal CV test sets for all parameter choices. For GM12878 F1 is only is slightly higher than random with chromosomal CV test sets. (TIFF) [file pcbi.1006625.s004.tiff]
